# Design and development of high precision four roll CNC roll bending machine and automatic control model

**DOI:** 10.1038/s41598-023-40204-7

**Published:** 2023-08-10

**Authors:** Yigang Jing, Shangsheng Jiang, Qun Sun, Ying Zhao, Zhihao Song, Xiangyan Meng, Hengzhen Li

**Affiliations:** School of Mechanical and Automotive Engineering, Liaocheng University, Liaocheng, 252000 China

**Keywords:** Engineering, Mechanical engineering

## Abstract

In recent years, advancements in industries such as aerospace, military weaponry, automobiles, locomotives, and shipbuilding have led to a surge in the demand for bent and rolled components, along with increasingly stringent requirements for rolling precision. However, the traditional hydraulic cylinder feeding solution has hindered further enhancements in the accuracy of rolled profile contours. Additionally, owing to variations in profile specifications, material properties, and an assortment of random factors during the forming process, the applicability of existing forming formulas remains limited, rendering them suitable only for profile processing under specific circumstances. To address these challenges, servo electric cylinders have been introduced as a replacement for traditional hydraulic cylinders, and the mechanical structure of a four-roll bending machine has been re-engineered. This innovation has demonstrated the feasibility of employing servo electric cylinders in four-roll CNC bending machines for profile bending, resulting in higher control precision and faster response times, ultimately providing a comprehensive design solution for four-roll CNC bending machines. In response to the limited universality of existing forming formulas, the actual R (profile forming curvature) and d (servo electric cylinder feed) values from the four-roll CNC bending machine have been utilized, and curve fitting methods have been implemented as the foundation for the automatic control model. This approach offers a high degree of universality, making it suitable for a wide range of applications. Moreover, as the number of trials increased, forming precision progressively improved.

## Introduction

Aluminum alloy profiles have been widely used in the aerospace field as one of the main load-bearing structures of aircraft frames due to their high specific strength, lightweight, and good formability^1,2^. Additionally, with the rapid development of coal power, hydropower, nuclear power, wind power, and petrochemical industries, there has been a focus on the design and development of roll bending machines and research on profile roll forming methods. Bending and forming processes are widely used in metal processing for various applications, such as in the oil and gas sector, naval sector, pipeline manufacturing, and automotive sector^3–6^. However, because roll forming involves both elastic and plastic deformation, there is elastic rebound, which can negatively impact the final forming accuracy and yield of the bent parts of the profile. The quality of bending and forming Z profiles, which are crucial components of aircraft fuselage brackets, can have a significant impact on the overall quality of the aircraft fuselage. The four-roller bending process has the advantages of high efficiency and low cost, and the parameters of the bending process play a crucial role in determining the profile bending quality.

Li^7^ found in the daily maintenance of the four high rolling mill that after years of operation and use, the lower mounted hydraulic system has exposed water ingress inside the hydraulic cylinder and some equipment problems caused by it, affecting the normal operation of production. The application field of electric cylinders is becoming increasingly widespread, and the market for electric cylinders is also growing^8^. Therefore, adopting the electric cylinder scheme is an experimental solution. In the research on the machine, Yue^9^ established the relationship between the force and bending moment and the driving power of the equipment, obtained a calculation method for the driving power of the main driving system of the four roll rolling machine, and selected the main motor power based on this calculation result. Huang^10^ utilized the finite element optimization design function of ANSYS software to simulate, analyze, and optimize the transmission components of the four roll rolling machine. Zheng^11^ conducted a theoretical analysis of the force deformation of the lower roller, derived a calculation formula for the force deformation of the lower roller, designed an effective compensation scheme, and used ABAQUS finite element analysis software to conduct three-dimensional dynamic simulation of the compensated state of the lower roller. Yao^12^ introduced the equipment and structural characteristics of a fully hydraulic four roll bending machine, with a focus on equipment composition, working principle, and design points. Yang^13^ fully understands the mechanical behavior during the pre bending process and analyzes the force acting on the working roller during the pre bending process of the four roll rolling plate. From the above, it can be seen that in the research on machines, some scholars focus on theory and calculation, while others focus on simulation and optimization. There is less experimental verification and machine construction for machines, so it is very important to replace traditional hydraulic and electro-hydraulic systems with servo electric cylinder systems and build machines.

In general, scholars have used a combination of the finite element method and experimental methods to analyze the impact of various factors on profile roll forming. However, there has been less research on CNC roll bending machines and their automatic control systems, with more research conducted on simpler three-roller models and less on more complex four-roller models. The hydraulic system used in current bending machines limits the forming accuracy, and the limitations of the forming method are very high. Based on this, this study verifies the rationality of the application of servo electric cylinders in a bending machine, and uses profile forming curve (R) and servo electric cylinder feed (d) values based on practical considerations as the basis of the automatic control model, which can be applied to products of any cross-section and material for bending.

## Four roll bending principle and motion relationship

### Four roll bending principle

**Figure 1:**
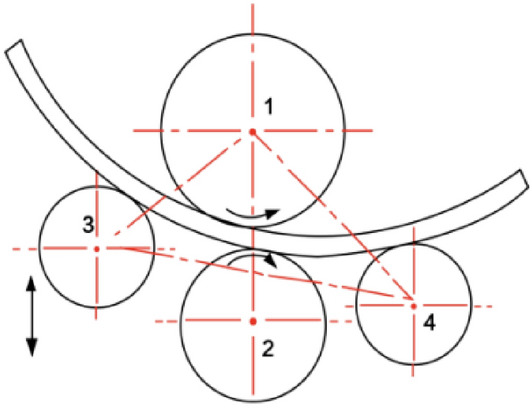
Roll bending principle.

The profile was placed in the middle of the upper and lower rollers, and the left and right lower rollers controlled the position of the rollers based on the rolling radius. As the rollers rotate, they drive the profile feeding and perform one or more feeding movements until the profile is processed into the desired curvature. The four-roller bending principle shown in Fig. 1 is designed to control profiles using an upper roller, and the friction generated between the profile and the upper roller propels the profile to complete the feeding movement and initiates the rotation of the other rollers. The upper and lower rollers were the active and driven wheels, respectively. The tangential force exerted by the upper and lower rollers on the sheet produces a driving moment that moves the profile in the tangential direction of the roller pointing to the profile displacement direction, whereas the left and right rollers move along the opposite direction of the tangential displacement direction and generate a frictional resistance moment in the tangential direction of the profile. At the beginning of the bending, the lower roller pressed the profile and held it between the upper and lower rollers, which ensured continuous and stable bending and deformation of the profile under the action of the frictional force of the rollers, effectively preventing the profile from being skewed or misaligned. After the lower rollers pressed the profile, the left and right rollers followed the path to complete feeding, and the profile was bent accordingly.

After the lower rollers pressed the profile, the left and right rollers followed the path to complete feeding, and the profile was bent accordingly.

### Mathematical model of machining process and initial displacement

**Figure 2:**
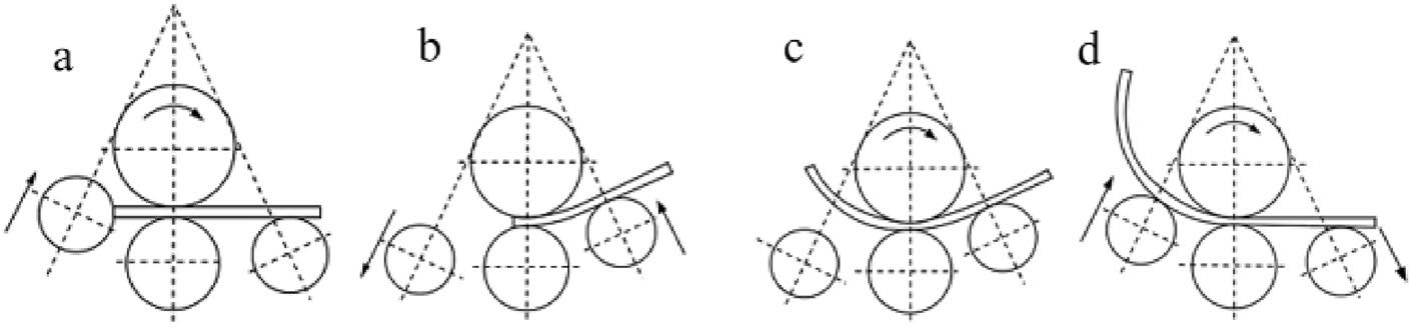
Roll bending process stages.

In the roll bending process, there is a certain center distance between the main and auxiliary rollers and the side bar, and the effect of roll bending can only be produced by the mutual extrusion of the main and auxiliary rollers with the largest center distance in the roll bending process. However, because of the existence of the center distance between the rollers, a part of both ends of the profile cannot be extruded with the largest center distance, so the problem of a straight line section will occur. This leads to profile wastage. To reduce profile waste, a pre-bending process was adopted. A process stages is shown in Fig. 2.

Centering is shown in Fig. 2a: by centering the profile before roll bending, it can be ensured that the end face of the processed profile is parallel to the side roll bus, thus effectively avoiding the distortion and deformation of the profile during rolling.

Pre bending is as shown in Fig. 2b, in the four roll symmetric bending machine, the asymmetric distribution of the roll positions can be used to pre bend the end of the part to reduce or even eliminate the residual straight edges, so that the resulting straight edge margin is 0. The main process of profile pre bending is as follows: first, on the basis of centering, the left roll descends to the corresponding position; second, the upper roll reverses to drive the profile back to the position with the corresponding margin on the left side of the profile; and finally, the right roll rises to the corresponding position, and the upper roll rotates in a positive direction to drive the profile to perform pre-bending.

The steady roll-bending process is shown in Fig. 2d, where continuous roll bending was the main process of the roll-bending forming. In this process, it is necessary to strictly control the speed and position of the upper roll and feed rate of the side roll. Based on pre bending, the right roll descends to the corresponding position, the left roll rises to the corresponding position, and the upper roll rotates in a positive direction to drive the profile feeding for continuous roll bending processing. The profile roll bending process after end-face centering, pre bending, and continuous roll bending is called pre bending side roll-lifting roll bending process.

**Figure 3:**
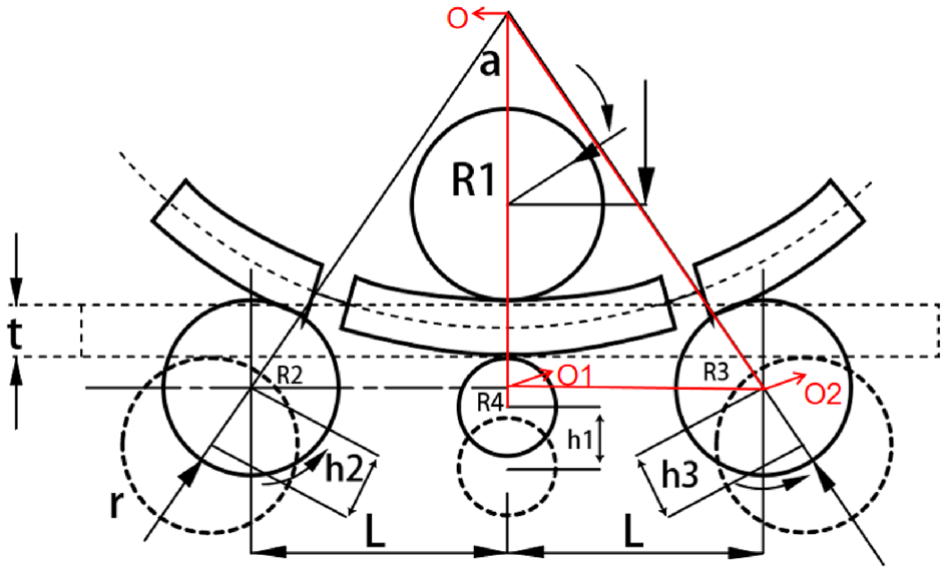
Analysis of Four Roll Bending.

As shown in Fig. 3, under the tightening action of upper roll R1 and lower roll R2, the forming curvature center of the pre bending roll bending section can be considered to be collinear with the upper and lower roll centers during four roll pre bending roll forming, and it is assumed that the curvature of the profile in the deformation zone is the same. It is assumed that the profile is always tangent to the straight edge during pre bending^14^.

According to the geometric model of the edge alignment process established in Fig. 3, the displacement of the lower roll is $h_{1}$, that of the left roll is $h_{2}$, and that of the right roll is $h_{3}$. According to the geometry, the displacement expressions of the lower, left, and right rolls can be obtained using (1). A schematic of edge alignment is shown in Fig. 2a:

1 $$ \left\{ {\begin{array}{*{20}c} {h_{1} = L_{1} - {\text{R}}_{1} - {\text{R}}_{2} - t} \\ {h_{2} = \frac{{{\text{h}}_{1} }}{\cos \alpha } + {\text{R}}_{3} } \\ {h_{3} = \frac{{{\text{h}}_{1} }}{\cos \alpha }} \\ \end{array} } \right. $$

The pre bending process, which is the preliminary work of profile roll bending, completes the pre bending of the left profile by raising (resetting) the right roll. The subsequent roll bending process is operated under the condition that the position of the pre bending work roll is basically unchanged; therefore, the work roll displacement of pre- bending and normal roll bending can be deduced together, and pre bending lifting can also be used as the single-side roll-lifting displacement formula,the pre bending process is shown in Fig. 2b.

It can be observed from Fig. 3 that the displacement of the lower roll is $h_{1}$, that of the left roll is $h_{2}$, and that of the right roll is $h_{3}$. According to the geometry, the displacement expressions for the lower, left, and right rolls can be obtained. Three points that are not on the same line determine a circle, ${\mathrm{OO}}_{1}$, ${{O}_{1}O}_{2}$, ${\mathrm{OO}}_{2}$ is three points that are not collinear, According to Pythagorean theorem, Formula (3) can be obtained. The location relationship of the three points is shown in Fig. 3.

2 $$\left\{\begin{array}{c}O{\mathrm{O}}_{1}=R+{\mathrm{R}}_{4}\\ {{O}_{1}O}_{2}={L}_{2}-{\mathrm{h}}_{3}sin\alpha \\ O{\mathrm{O}}_{2}=\left(\mathrm{R}+{\mathrm{R}}_{2}+{\mathrm{R}}_{4}-{\mathrm{R}}_{2}-{\mathrm{h}}_{3}\mathrm{cos\alpha }+{\mathrm{h}}_{1}\right)\end{array}\right.$$

3 $${\left({\mathrm{OO}}_{1}\right)}^{2}+{\left({{O}_{1}O}_{2}\right)}^{2}={\left({\mathrm{OO}}_{2}\right)}^{2}$$

### Key technologies of the equipment

During the development of the roll bending machine, various roll bending machines available in the market were investigated, and the technical characteristics of the machine were innovatively proposed by analyzing the advantages and disadvantages of different machines. A four-roller feed was selected over a more mature three-roller structure. Four-roller roll bending can reduce the straight edge margin compared with three-roller roll bending, but it increases the control difficulty. The use of servo-electric cylinders instead of traditional servo-cylinders provides a higher control accuracy, helps to improve the accuracy of the profile, and reduces the size of the machine. The left and right lower rollers of the machine are driven by servo-electric cylinders, with a position control accuracy of ± 0.01 mm and high repeatability accuracy to ensure the consistency of the profile processing, and the fast response of the servo-electric cylinder position control ensures the accuracy of the profile bending. Spherical bearings are used at the push-rod connection, which can withstand radial loads, axial loads, or combined radial and axial loads simultaneously. The rollers were mounted on a specially designed T-joint table to ensure smooth feeding and machining, and an integrated bed was used to perform profile bending and rounding more smoothly. By integrating the VS software with the GTS-800 motion controller, a PC can be transformed into a motion controller with real-time motion-control capability. The actual R and d values of the four-roller CNC bending machine are utilized, and a curve fitting method is employed to select a basic curve equation as the foundation for the control model in the automatic control design. To sum up, it can be seen as a major improvement and upgrading of the four-roll bending machine.

### Control mechanism position relationship

**Figure 4:**
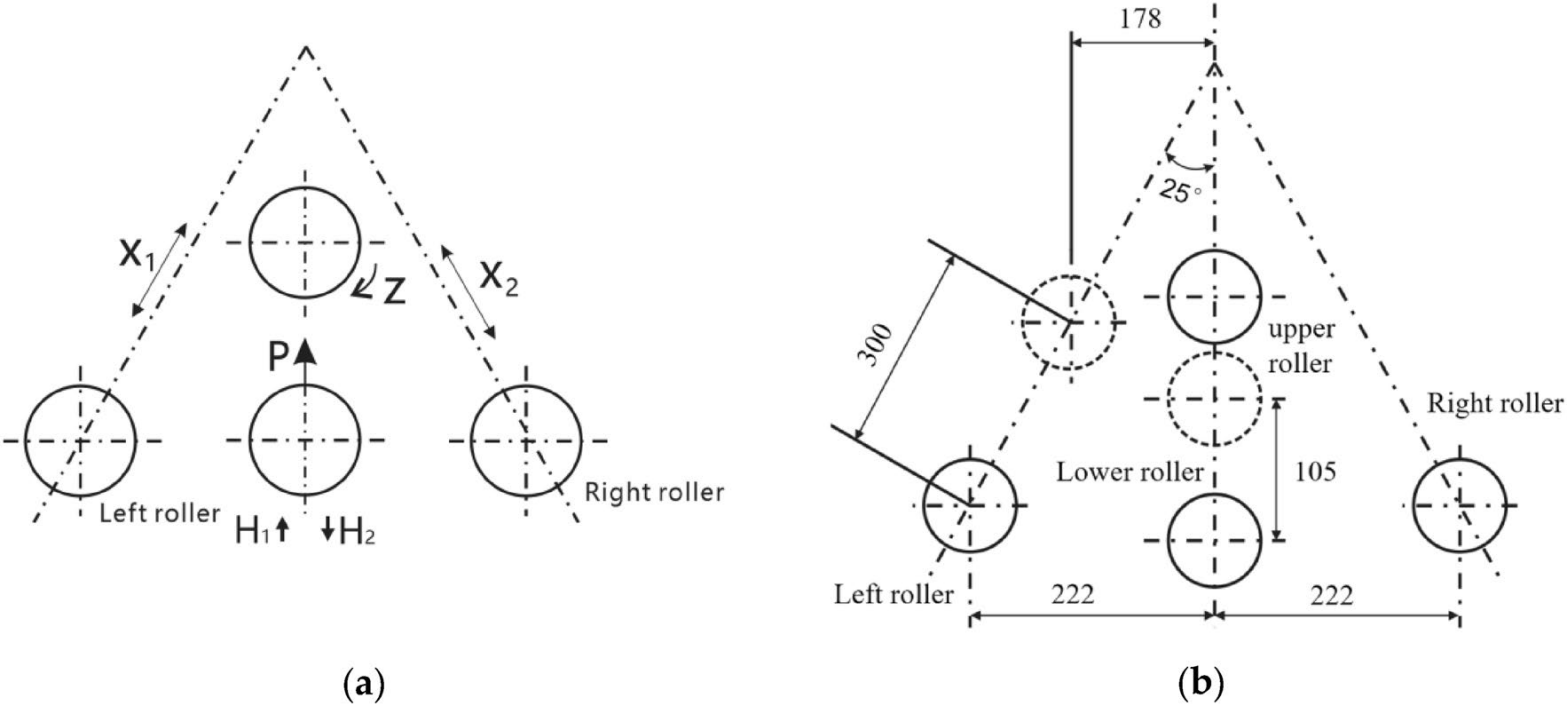
(**a**) Motion system; (**b**) Position relationship of control structure.

The motion system is illustrated in Fig. 4a, which has three motion coordinate parameters that can be numerically controlled, that is, a rotational main motion and parameters for the synchronous control of movement in two directions.

1. Z-axis: main spindle for clamping and conveying the workpiece;
2. X–Y-axis movement: used for bending and forming the profile.
3. P-axis movement: This provides pressure to clamp the workpiece.
4. U-axis movement: closing and opening the lower rollers.

The upper roller is a fixed wheel that mainly serves as the driving and guiding component. The lower roller was a clamping wheel that could move up and down, and the clamping pressure P could be numerically controlled. The left and right rollers work together to complete radial bending, with the directions of movement of the left and right rollers shown in the X1 and X2 directions, respectively.

The geometric relationship and dimensions between the control structure positions of the CNC four-roller roll-bending machine designed and manufactured in this study are shown in Fig. 4b. The dashed-line positions of the rollers in the figure represent the maximum travel position during their movement.

## Key component selection and calculation.

### Bending force analysis

**Figure 5:**
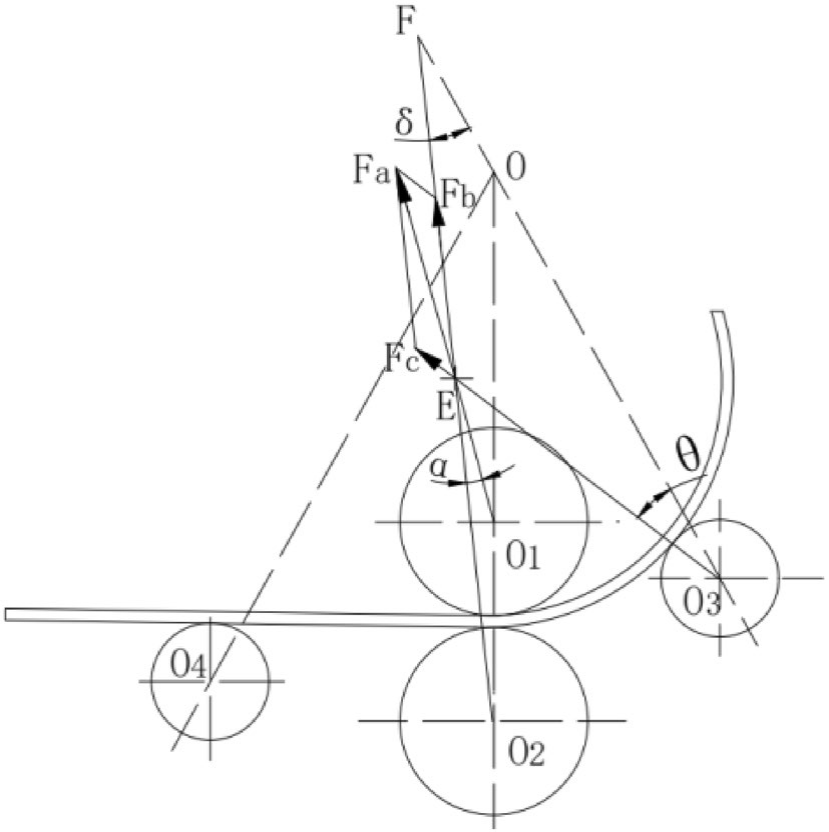
Bending force diagram of profile.

A stress diagram of the continuous rolling-bending process of the profile is shown in Fig. 5.

Force acting on the left roller $F_{c}$

4 $$ F_{c} = \frac{M}{{\left( {R + t} \right)\sin \alpha }} $$

Force acting on the lower roller $F_{b}$

5 $$ F_{b} = \frac{M}{{\left( {R + t} \right)\sin \left( {\delta + \theta { - }\alpha } \right)}} $$

Force acting on the upper roller $F_{a}$

6 $$ F_{a} = \frac{M}{{\left( {R + t} \right)}}\left( {\frac{1}{\tan \alpha } + \frac{1}{{\tan \left( {\delta + \theta - \alpha } \right)}}} \right) $$

### Calculation of bending moment of metal profiles

The stress distribution on the plate section along the steel plate height during linear pure plastic bending and the functional relationship of the true stress can be expressed as follows:

7 $$ \sigma = \sigma_{s} + E_{2} \varepsilon $$

where $\sigma$ is the stress of the workpiece,$\sigma_{s}$ is the yield limit of the material, $\varepsilon$ is the strain of the workpiece,and $E_{2}$ is the linear strengthening modulus of the material, which can be obtained by referring to the relevant manuals.

The bending moment M on the section is:

8 $$ M = \int_{A} {\sigma_{y} } dA = 2\int_{0}^{{\frac{\delta }{2}}} {\sigma bydy} $$

where b is the maximum width of the steel plate rolled by the plate-rolling machine. The initial bending moment of deformation $M_{0}$ is

9 $$ M_{0} = \sigma_{s} \frac{{b\delta^{2} }}{4} $$

### Driving torque of upper roll

The upper roll of the four-roll plate rolling machine was the driving roll. The total driving torque acting on the upper roll is the sum of the torque consumed during deformation and the torque required to overcome the friction. The friction torque includes the friction resistance consumed in overcoming roller rolling on the bending plate, and the friction torque consumed in the roller bearing.

The torque consumed during deformation can be determined using the condition that the work done by the bending internal force is equal to the work done by the external force applied to the upper roll:

10 $$ W_{n} = \frac{{M_{0} + M}}{2} \cdot \frac{L}{{R{\prime} }} $$

11 $$ W_{w} = M_{n1} \cdot \frac{L}{{{{D_{a} } \mathord{\left/ {\vphantom {{D_{a} } 2}} \right. \kern-0pt} 2}}} $$

where $W_{n}$ is the work done for bending the internal force, $W_{w}$ is the work of the external force acting on the upper roll, and $L$ is the length of the plate corresponding to the bending angle.

Let formulas (10) and (11) be equal. The torque consumed during deformation can be written as

12 $$ M_{0} = \sigma_{s} \frac{{b\delta^{2} }}{4} $$

The torque required to overcome friction can be determined using (13). Friction torque when shaft rollers are arranged asymmetrically

13 $$ M_{n2} = f\left( {F_{a} + F_{b} + F_{c} } \right) + \frac{\mu }{2}\left( {F_{a} d_{a} + F_{b} d_{b} \cdot \frac{{D_{a} }}{{D_{b} }} + F_{c} d_{c} \cdot \frac{{D_{a} }}{{D_{c} }}} \right) $$

where $f$ is the rolling friction coefficient, take $f = 0.8\;{\text{mm}}$; $\mu$ is the sliding friction coefficient at the journal, and $\mu = 0.05\sim 0.1$ $d_{a}$, $d_{b}$, $d_{c}$ are journal diameters of the upper roll, lower roll, and side roll, respectively.

The total driving torque acting on the upper roller is:

14 $$ M_{n} = M_{n1} + M_{n2} $$

### Upper roller drive power

The calculation formula for driving power is:

15 $$ P = \frac{{M_{n} v}}{60r\eta } $$

where $v$ denotes the profile speed, $r$ denotes the driving roller radius,take $r = {{D_{a} } \mathord{\left/ {\vphantom {{D_{a} } 2}} \right. \kern-0pt} 2}$; $\eta$ denotes the transmission efficiency.

### Servo electric cylinder

#### Principle of servo-electric cylinder.

The servo electric cylinder is a novel modular product that integrates the design of a servo motor and a ball or roller screw. By converting the rotational motion of the servo motor into linear motion, this product enables precise control over the speed, rpm, and torque. Furthermore, the advantages of precision control of the servo motor can be leveraged to achieve precise speed, position, and thrust control, making this product a revolutionary solution for high-precision linear motion applications.

#### Features of servo-electric cylinder

1. The utilization of servo motor control in the servo electric cylinder system allows for precision control of thrust, speed, and position by leveraging the closed-loop control characteristics of the servo motor.
2. The electric cylinder presents several advantages over traditional hydraulic cylinders, such as improved response reliability, increased precision stability, and advanced control features.
3. The servo electric cylinder is characterized by prolonged service life, exceptional environmental adaptability, and robust start-stop capabilities. Through the integration of servomotor and electric cylinder technologies, this novel product can achieve superior environmental performance, energy efficiency, and high-precision motion control capabilities.

#### Structure of servo-electric cylinder

**Figure 6:**
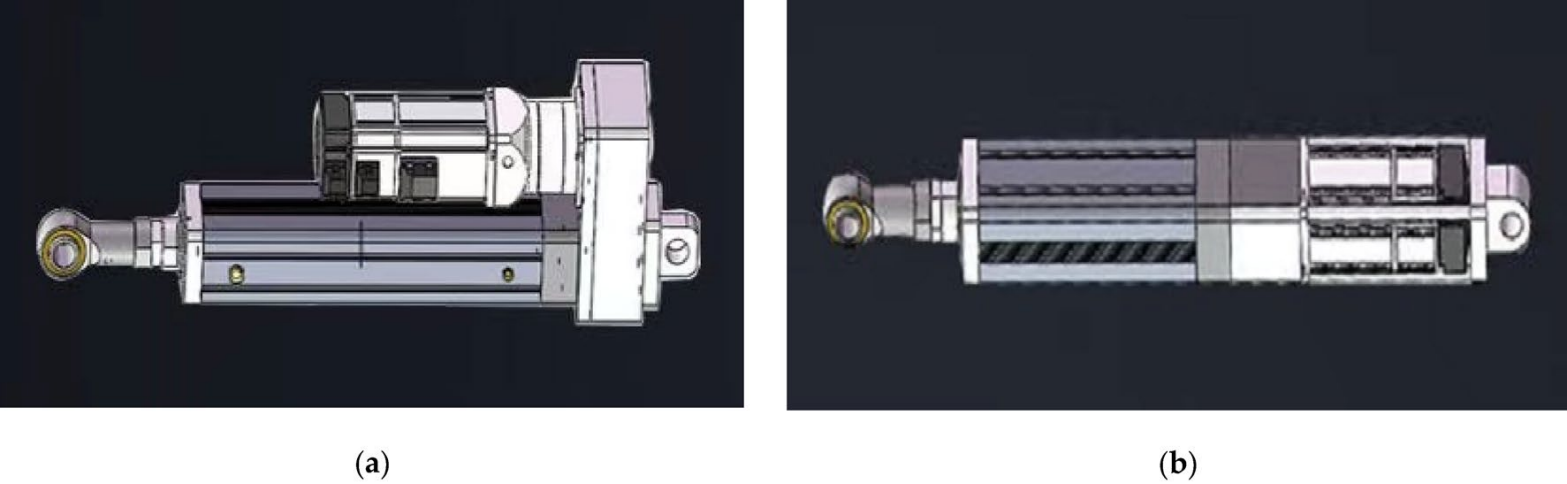
(**a**) Parallel servo electric cylinder. (**b**) Series servo electric cylinder.

The structure of the servo electric cylinder is relatively simple, mainly composed of four parts: driving mechanism, reduction device, linear transmission mechanism, and transmission mechanism. There are two structural forms of servo motors and electric cylinders. One is the direct connected electric cylinder, as shown in Fig. 6a. The direct connected servo motor is directly connected to the motor through a coupling. Another type is the parallel structure, as shown in Fig. 6b, where the electric motor is installed in parallel with the electric cylinder through a high-strength synchronous belt, also known as a parallel structure or a foldable structure. In addition to the characteristics of series electric cylinders, the total length of parallel electric cylinders is also relatively short, making them more suitable for installation in limited space.

#### Study on the selection of servo-electric cylinder

The selection of suitable servo-electric cylinder parameters is primarily dependent on various factors, such as cylinder load, cylinder life, number of cycles, travel distance, and installation space.

1. Load
  Accurate knowledge of the load is essential for identifying the most cost-effective and dependable electric-cylinder solution.
  The relationship between the output torque of the motor and the output force of the electric cylinder is
  16 $$ F = \frac{T \cdot \eta \cdot 2\pi \cdot R}{L} $$
  where $F$ is the output force of the electric cylinder, $T$ is the motor output torque, $R$ is the transmission ratio, $L$ is the lead of the lead screw (mm), and $\eta$ is the mechanical efficiency, typically 85–90%.

Equation (17) can be used to initially estimate the motor specifications required to satisfy the load demands. Along with the maximum force exerted during operation, the force variation throughout the stroke is also a critical factor to consider. The average load, which can be derived from the force variation throughout the operating cycle, served as the basis for calculating the service life of the cylinder.

The average load calculation formula of the electric cylinder is:

17 $$ F_{m} = \sqrt[3]{{\frac{{F_{1}^{3} \cdot V_{1} \cdot t_{1} + F_{2}^{3} \cdot V_{2} \cdot t_{2} + F_{3}^{3} \cdot V_{3} \cdot t_{3} }}{{V_{1} \cdot t_{1} + V_{2} \cdot t_{2} + V_{3} \cdot t_{3} }}}} $$

**Figure 7:**
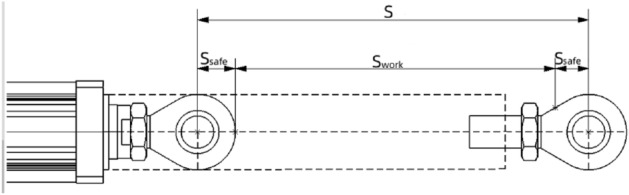
Working distance of electric cylinder.

(2) Lifetime.
  In the context of electric cylinders, the term "life" typically pertains to the lifespan of the screw employed within the cylinder. This lifespan can be subdivided into two distinct components: the fatigue life of the screw, which can be quantified through calculation, and the service life, which is contingent on various usage conditions including temperature, average load, lubrication type, frequency of lubrication replenishment, and other relevant factors.
  The service life calculation formula is.
  18 $$ L_{10} = \left( {\frac{{C_{a} }}{{F_{m} }}} \right)^{3} L $$
  where $L_{10}$ is the service life of the electric cylinder,$C_{a}$ is the basic rated dynamic load of the screw road surface,$F_{m}$ is the average load borne by the electric cylinder, and L is the lead load of the lead screw.
(3) Cycle Count.
  By accurately specifying the acceleration and speed of the actuator or providing the cycle time and required distance, it is possible to select an optimal electric cylinder.
(4) Running Distance and Installation Space.
  The selection of the electric cylinder, including its working stroke and installation space, is highly interrelated. During operation of an electric cylinder, it is imperative to ensure that it does not reach the end of its mechanical limit. Therefore, a safety stroke must be incorporated at both ends of working stroke S, resulting in a longer section of the stroke. The sum of these two strokes constitutes the running distance S of the electric cylinder, that is,$S = S_{{{\text{work}}}} + 2S_{{{\text{safe}}}}$, as illustrated in Fig. 7. Depending on the installation space requirements, a series- or parallel-structure electric cylinder can be selected.

### Complete machine assembly

**Figure 8:**
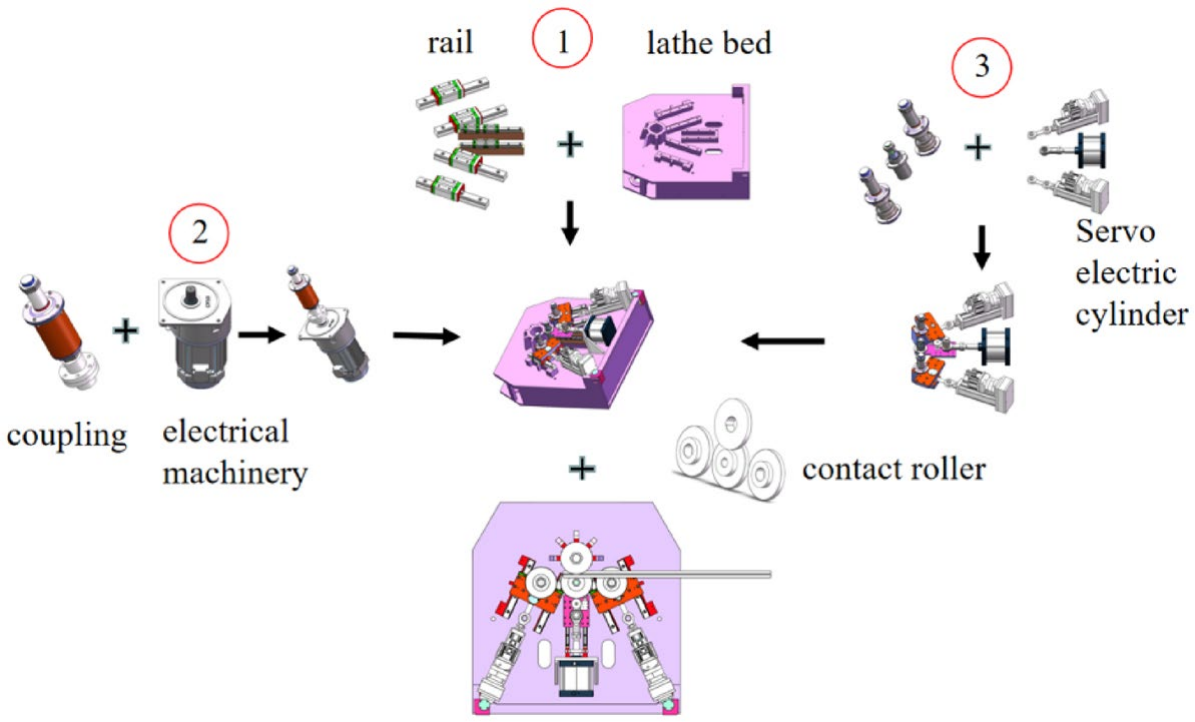
Complete machine assembly process.

Figure 8-1 shows the installation of the slider guide rail on the bed, Figure 8-3 shows the installation of the spindle motor and coupling, and Figure 8-3 shows the installation process of the servo cylinder and side roller shaft, which is finally installed as a whole machine.

**Figure 9:**
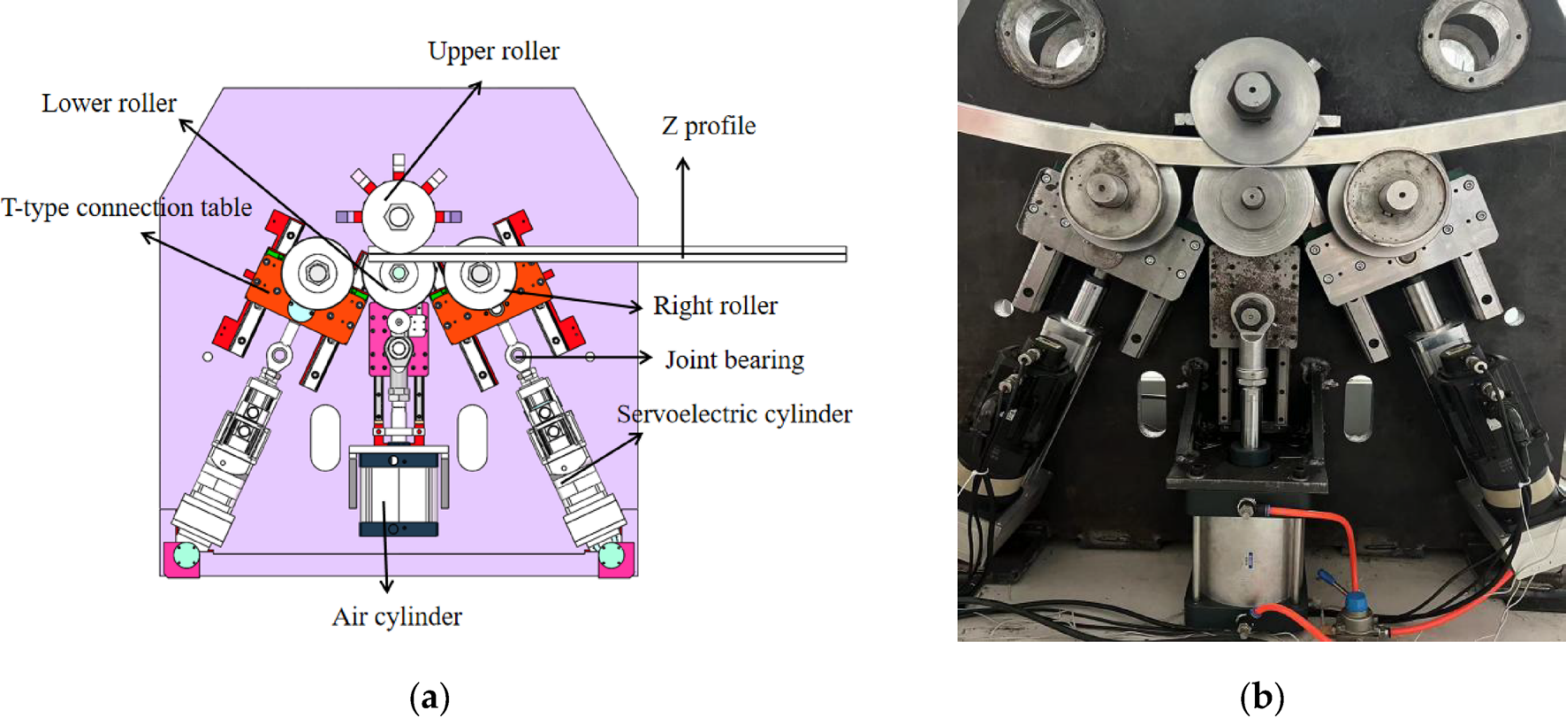
Four roll CNC bending machine (**a**) the model; (**b**) the actual machine.

The roll-bending machine consists of three main parts: the mechanical equipment, pneumatic system, and CNC system. The mechanical structure of the machine is comprised of four rollers, including the upper rollers, left- and right-side rollers, lower center rollers, servo electric cylinder, spindle motor, and frame. The upper roller is fixed on the frame, while the left- and right-side rollers are driven by servo electric cylinders to perform the feeding movement along the guide rail. A three-dimensional model of the four-roller CNC roll-bending machine is shown in Fig. 9a, and the actual object is shown in Fig. 9b.

### Discuss

**Figure 10:**
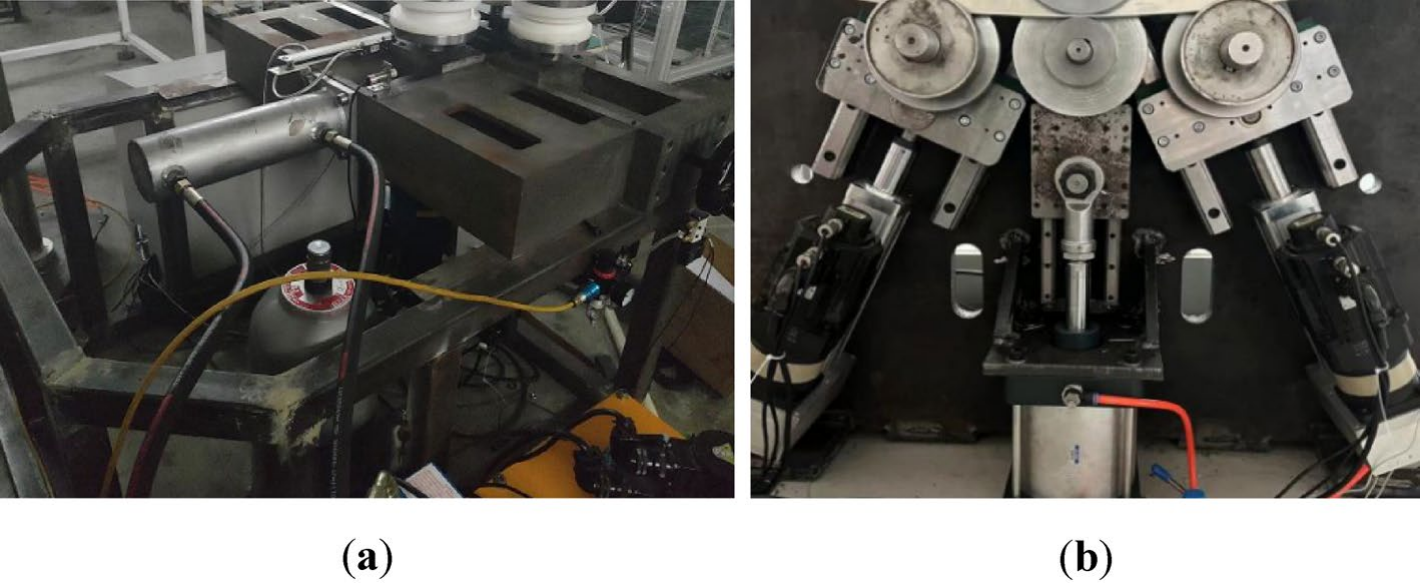
(**a**) Hydraulic system controlled roll bending machine ; (**b**) Roll bending machine with servo electric cylinder control scheme.

From the comparison between Fig. 10a and b, it can be found that the mechanical structure using the servo electric cylinder scheme is cleaner, and the use of pure electric control increases the safety of the machine during operation. The specific comparative analysis is shown in the Table1.

**Table 1:** Comparative analysis of hydraulic system and servo electric cylinder system.

	Hydraulic cylinder	Servo electric cylinder
Transmission medium	Hydraulic oil	Mechanical structure
operation temperature	The working temperature range of hydraulic cylinders is usually set at − 40 to 120 °C, and their performance is easily affected by temperature fluctuations	The operating temperature range of the electric cylinder is usually set at − 30 to 80 °C and its performance is less affected by temperature fluctuations
structure	Requires hydraulic pumps, hydraulic valve groups, and hydraulic pipelines, which occupy a large space and have a complex structure. Fault diagnosis and troubleshooting require high technical skills	Requires electric motors and mechanical transmission components, occupies small space, is easy to arrange, and has a simple structure
Position Controllability	Difficulty in position control	Easy position control, capable of achieving precise positioning of around 0.01 mm
Transmission efficiency	Less than 50%	90%

## Overall design of the control system plan

**Figure 11:**
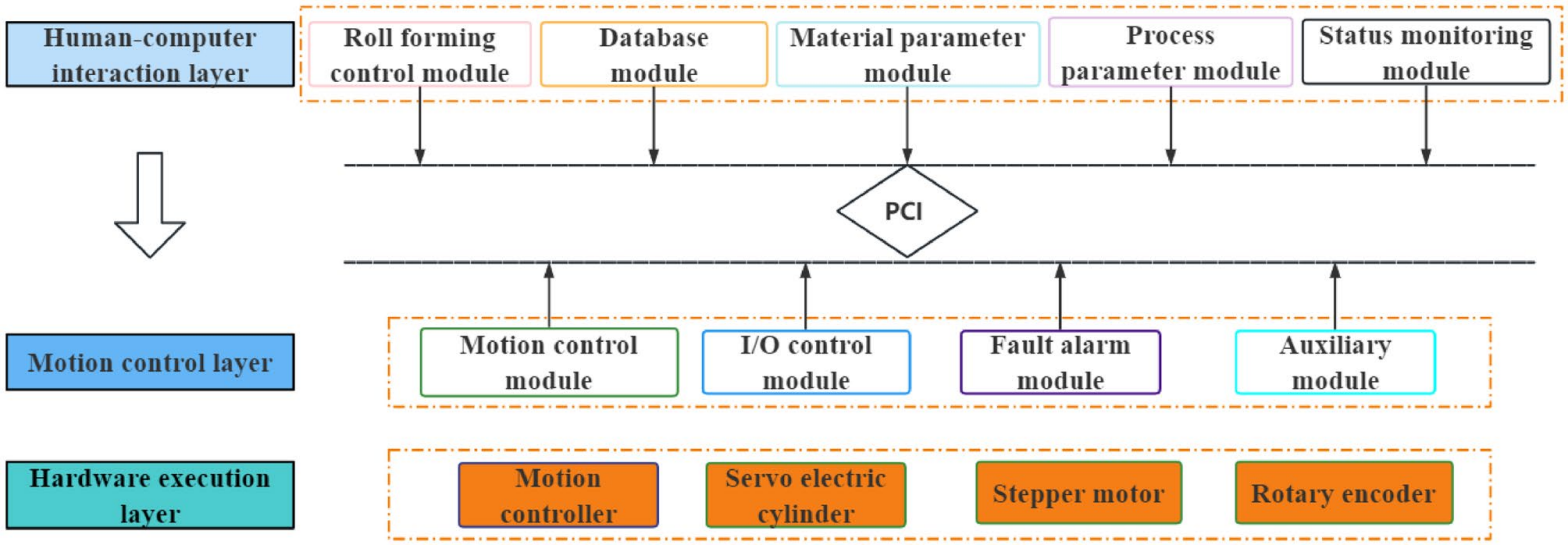
Control system scheme.

Based on the requirements of the control system of the CNC four-roller bending machine, a Programmable Logic Controller motion control card system solution was adopted with a hierarchical and modular design approach, as illustrated in Fig. 11. This control system solution is an open architecture with portability and tailorability built under the Windows Forms application module of Microsoft Visual Studio software (.NET Framework). The Windows Forms application module provides the necessary functionality to write a complete motion-control solution, including the host computer interface. The GTS-800-PV-PCI series of PC-embedded motion controllers from Goodco was chosen for this control system owing to its ability to enable high-speed point-position motion control. The core of the controller consists of a DSP and an FPGA, which enables high-performance control calculations. GTS-800 is widely used in a range of applications such as robotics, computer numerical control (CNC) machine tools, woodworking machines, printing machines, assembly lines, electronic processing machines, laser processing machines, and PCB drilling and milling machines. By combining the Visual Studio software with the GTS-800 motion controller, the PC can be turned into a motion controller with real-time motion-control capability. The Visual Studio software completes the motion control function, replacing traditional hardware control, thereby breaking the limitation of traditional hardware incompatibility and making the control system more open and easy to expand its functions to meet diversified needs. The system can also use various resources in the Windows system, making it more modular, hierarchical, and standardized while shortening the development cycle of customized products. Microsoft Visual Studio (VS) is a development toolkit series product from Microsoft Corporation in the United States. VS is a basically complete set of development tools, which includes most of the tools needed in the entire software life cycle, such as UML tools, code control tools, Integrated development environment (IDE), and so on.

**Figure 12:**
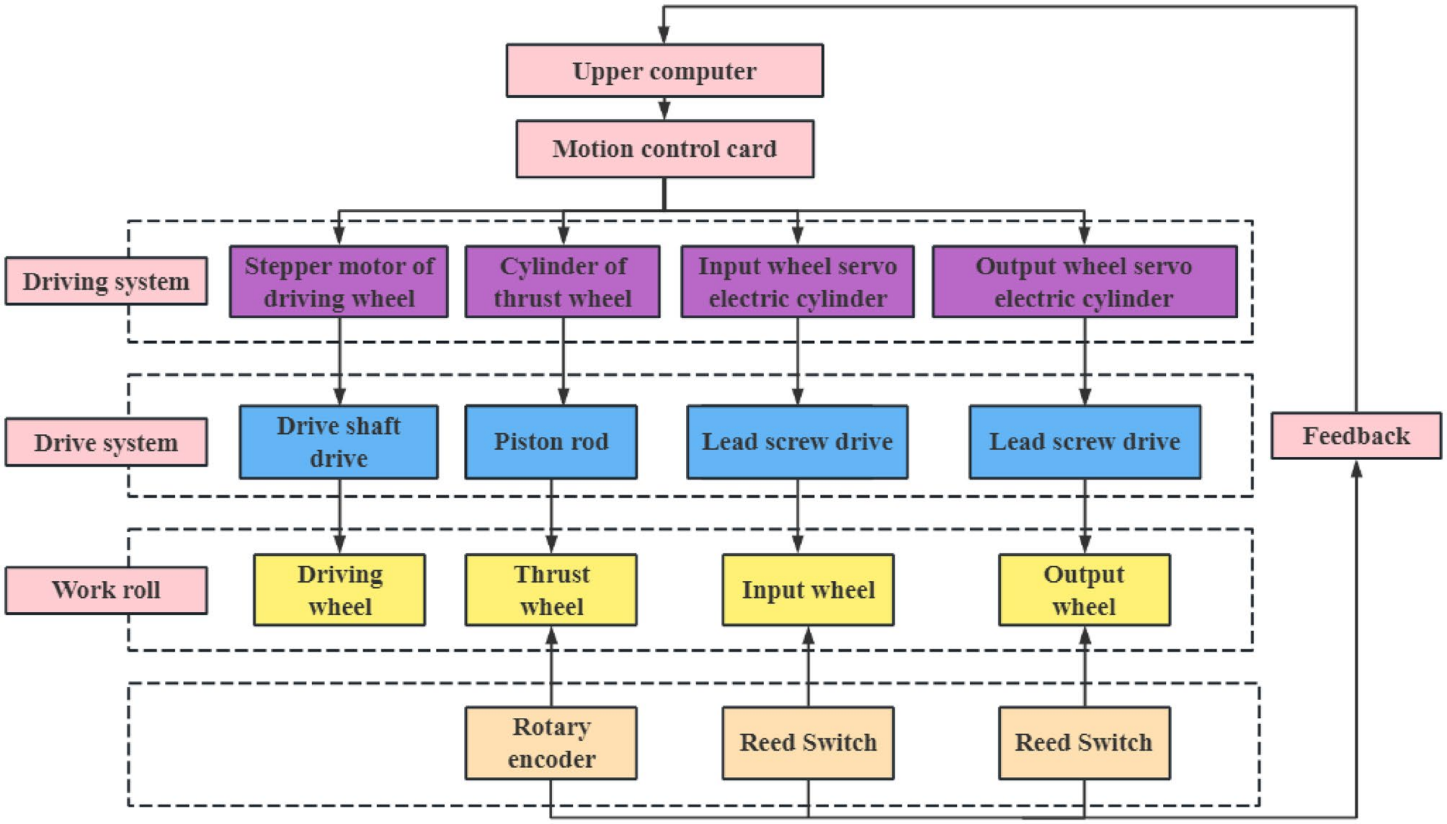
Control system principle.

According to the control process of the roll-bending machine, a schematic diagram of the control system is designed, as shown in Fig. 12.

## Simulation verification

### Profile and roller dimensions

**Figure 13:**
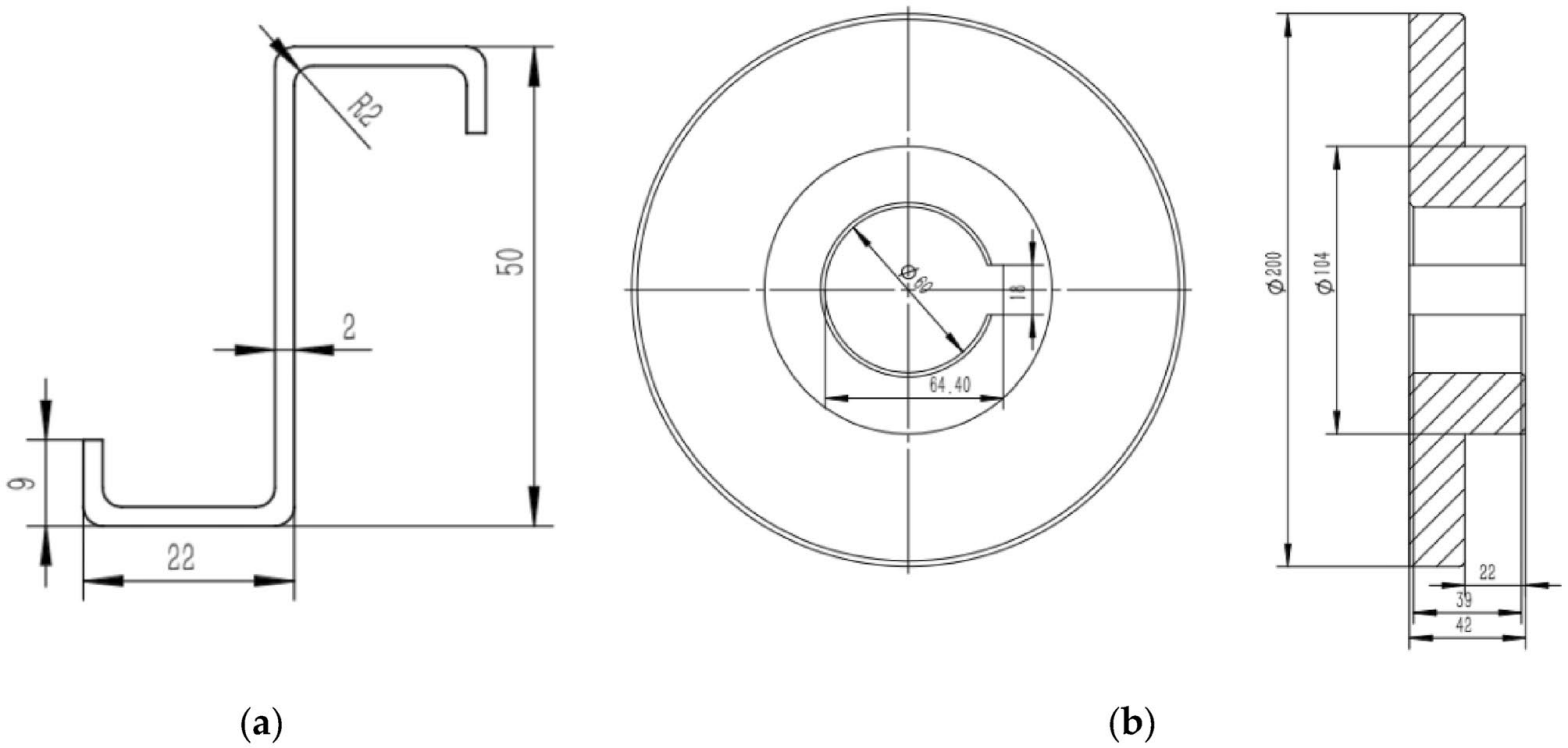
(**a**) Z profile section ; (**b**) 4-roller CNC rounding machine. contact roller.

To achieve a lightweight design of the aircraft, the new large aircraft uses large cross-section Z profiles to form frame edge type parts, which are mainly produced using the roll bending process^12^. Aluminum alloy profiles, an important part of fuselage support, require processing into bent parts with a certain bending shape to meet the support of the fuselage streamlined skin. Thus, studying the bending and forming process of a profile is significant for improving aircraft manufacturing capability. Z-profile bent parts, as an important part of the aircraft fuselage support, are usually rolled and bent from a Z-profile straight material with a thickness of 2 mm and a length of 1300 mm, as shown in Fig. 13a. Based on this section data, the roller involved is shown in the figure, and the size of the roller is shown in Fig. 13b.

### Establishment of finite element model for four-roll bending

The finite element simulation method has the advantages of a short test cycle and low cost. At the same time, the laws and relationships among variables in the forming process can be analyzed more accurately and have been widely used in various fields of profile roll bending analysis^15^, which is considered to be the most effective method for profile bending research^16,17^. Based on this, the finite element software ABAQUS was used to establish a dynamic finite element model of four-roll roll bending forming to verify whether the designed roller position can bend the profile.

#### Build geometric model

**Figure 14:**
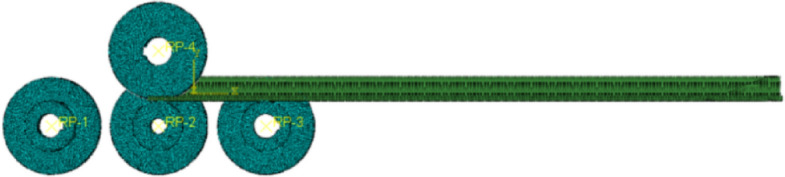
Finite element model of four-roll roll-bending profile.

The four-roll bending machine was designed and manufactured independently using ABAQUS to establish a finite element model of four-roll profile bending. It is known that the roll (large) diameter is 200 mm, the roll (small) diameter is 104 mm, the side roll inclination is 25°, and the deformation of the roll is small and negligible; therefore, it is defined as discrete rigid. The elastic–plastic deformation of the profile during the rolling process must be defined as deformable. A finite element model of the four-roll rolling profile is shown in Fig. 14.

#### Material properties

In this simulation experiment, a four roll bending simulation was conducted using 6063-T5 aluminum alloy as the profile. It was assumed that the sheet material was isotropic, and the influence of the sheet weight on roll bending was not considered. Elastoplastic deformation of the profile occurs during roll bending; therefore, the constitutive relation of the profile must be set in ABAQUS. As the bilinear hardening model can better fit the real constitutive relationship of materials, In this study, the bilinear hardening model is used to define the Constitutive equation of the profile, which is used to simulate the plastic deformation of the profile in the process of rolling and bending. The constitutive equation is given by (19). 6063-T5 is the grade of aluminum alloy.

19 $$\left\{\begin{array}{c}{E}_{\varepsilon }, \quad 0<\left|\varepsilon \right|<{\varepsilon }_{e}\\ {\sigma }_{s}+K\left(\varepsilon -{\varepsilon }_{e}\right), \quad \left|\varepsilon \right|>{\varepsilon }_{e}\end{array}\right.$$

where σ is the stress, ε is the strain, and E is the Young's modulus.

#### Contact definition and constraint

The penalty function contact mode was adopted, the friction coefficient between the roller and profile was defined as 0.5, and the roller and sheet were defined as having a limited relative slip. When the profile is continuously rolled and bent, the upper and lower rollers can rotate only around their respective axes. The side rollers can not only rotate around their axes but also feed along a fixed oblique line (see Table 2 for constraint types).

**Table 2:** Constraint types of each part of roll-bending form.

Constraint type	Upper roller	Lower roller	Left roller	Right roller
Move constraint	X, Y, Z	X, Y, Z	X	X
Rotational constraint	Y, Z	Y, Z	Y, Z	Y, Z

### Analogue simulation

**Figure 15:**
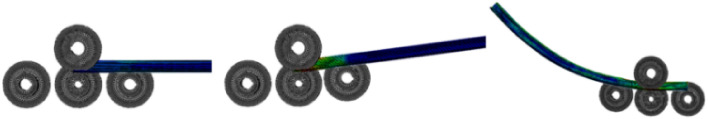
Profile rolling process.

The finite element model is established according to the design data, and the roll bending process of the profile is simulated to verify whether this structure can meet the profile bending. The profile bending process is shown in Fig. 15, which can clearly see that the profile can be well bent, and this design structure has certain rationality.

## Automatic control mathematical model

Bending formation is a type of plastic deformation. During unloading, the outer fiber is shortened owing to elastic recovery, and the inner fiber is extended owing to elastic recovery. Consequently, the curvature and angle of the bending part changed significantly. This phenomenon is known as spring-back effect. spring-back is a mechanical property of materials during deformation; therefore, it cannot be eliminated, and we can only try to control the amount of spring-back effect^18^. spring-back effect is inevitable in the actual roll bending operation of the profile. Owing to its special section structure, the profile inevitably exhibits non-uniform deformation during the roll bending process, resulting in more obvious springback^19^. Accurate control of springback is a difficult problem.

Owing to the different profile specifications, uncertainty of material properties, and role of various random factors in the forming process, a large error in deriving the forming formula may occur^20^. Therefore, this study considers the actual R and d values of the four-roller CNC round-bending machine and adopts a simple curve fitting method to select a simple curve equation as the basis for the control model in the automatic control design, which serves as the initial verification of the profile roll forming accuracy.

Many factors affect the springback of profiles, including the Young's modulus, yield limit, profile thickness, profile weight, and friction between the profile and roller. Therefore, it was difficult to establish a high-precision springback model. In engineering, combining theory and experience, the commonly used plate rebound model^21^ is transformed into:

20 $$\mathrm{R}=\frac{1-{\mathrm{K}}_{0}{\upsigma }_{\mathrm{s}}/\mathrm{E}}{1+2{\mathrm{R}}_{\mathrm{f}}{\mathrm{k}}_{1}{\upsigma }_{\mathrm{s}}/(\mathrm{ET})}{\mathrm{R}}_{\mathrm{f}}$$

However, in the experiment, it was found that formula (20) is more suitable for profiles processed with Q235 steel, and the testing error for other materials is relatively large. For the 6063T-5 aluminum alloy profile studied in this article, the error exceeds 100% and cannot be used to guide production and processing. Therefore, this study considers establishing an adaptive control model that can be applied to various materials and cross-sections from a practical perspective.

**Figure 16:**
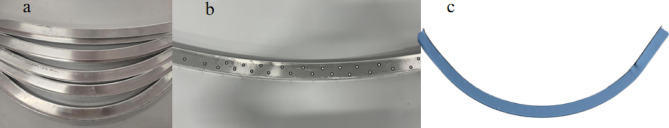
(**a**) Test profile (**b**) Reflective patch on profile(c) Laser scanning 3D model.

Rolling experiments were conducted on the 6063T-5 aluminum alloy, and the roll bending profile is shown in Fig. 16a. Second, the processed profiles were pasted with reflective stickers, as shown in Fig. 16b. Finally, the processed profile was scanned and modeled using a CREAFORM portable metrological 3D scanner, as shown in Fig. 16c. The curvature of the processed profile is obtained by using the reverse expansion idea to ensure the accuracy of the profile curvature. Therefore, the following Eq. (21) can be used to represent the rolling forming curve. Carry out statistical regression analysis on the experimental results, We found that the R–d forming curve obtained from the experiment matched well with a power function equation.

21 $$\mathrm{d}={\mathrm{aR}}^{\mathrm{b}} (\mathrm{b}<0)$$

In the formula: d—Side roll displacement, mm; R—Profile forming radius, mm; a—coefficient; b—power constant(b < 0).

Using the selected Z-section data as an example, the four-roll CNC bending machine had a large roller diameter of 200 mm and a small roller diameter of 104 mm, as shown in Fig. 15. The test material was a 6063-T5 aluminum alloy, and its dimensions are shown in Fig. 14. The R and d values for each rolling step are listed in Table 3.

**Table 3:** R, d experimental data of roll bending.

Order number	Side roll displacement d (mm)	Forming radius R (mm)
1	20	17,873.28
2	23	15,269.76
3	25	14,749.16
4	27	12,337.63
5	30	10,021.40
6	33	9367.36
7	38	7758.35
8	40	7341.803
9	43	6638.45
10	45	6344.59
11	47	5751.45
12	50	5105.64

Through regression calculation, the power function equation of the processing system is:

22 $$\mathrm{d}=1007176.03192{\mathrm{R}}^{-1.33693}$$

**Figure 17:**
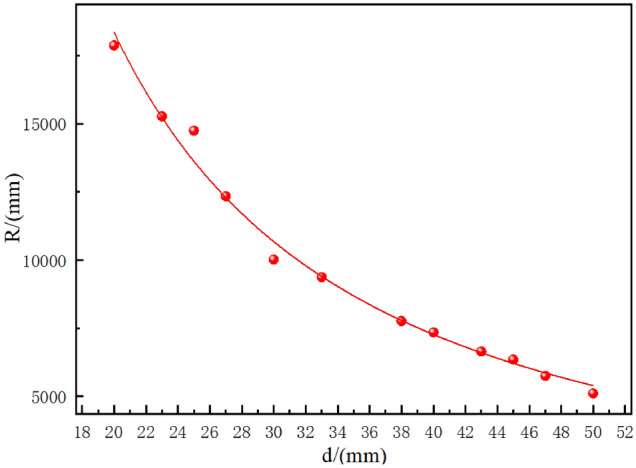
R–d forming curve.

**Figure 18:**
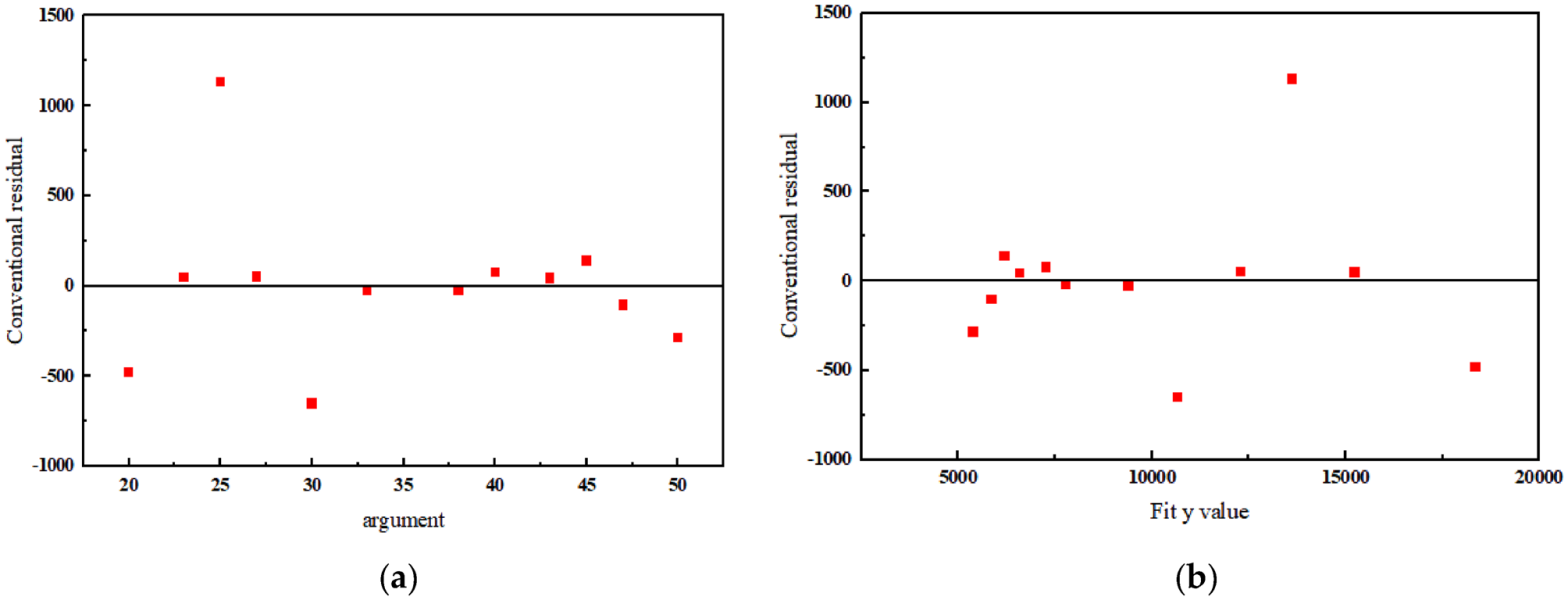
R–d forming curve.

According to the data in Table 2, the fitted Coefficient of determination $R^{2} = 0.98955$, the greater the r, the better the model fitting effect; The closer r is to 1, the better the regression effect. Therefore, the relationship between side roll feed d and sheet forming radius r follows the rule of Power function curve when the profile roll bending experiment is carried out on a four high NC mill. The R–d forming curve of this experiment is shown in Fig. 17. The fitting residual plot is shown in Fig. 18.

To reduce profile waste, two sets of experimental values were selected and substituted into Eq. (22). Three experiments were conducted, and the results are listed in Table 4.

**Table 4:** error analysis.

Order number	Side roll displacement d (mm)	Forming radius R (mm)	Curve fitting (mm)	Error (%)
1	43	6638.45	6595.87	0.6
2	47	5751.45	5865.35	1.98
3	52	5189.36	5115.98	1.41
4	55	4874.25	4746.37	2.62
5	57	4412.16	4525.05	2.56

The average error of the five groups of tests is 1.834%, which can be used for preliminary processing of profiles. As the number of tests increases, accurate processing of profiles can be achieved. According to the degree of dispersion, it is found that the error is relatively large when the side roller is fed 25 mm, and the error between the experimental value and the curve fitting value is 7.1%.

To verify the effect of the number of experiments on the forming accuracy, six sets of data were selected from Table 2, namely 1–6,11–7,11–8,11–9,11–10,1–12, and fitted separately. The results of data fitting are shown in Table 4. The fitted results are shown in Table 5.

**Table 5:** Fitting coefficient.

order number	Number of groups	Fitting coefficient
1	1–6	0.96441
2	1–7	0.97589
3	1–8	0.98129
4	1–9	0.98482
5	1–10	0.987
6	1–11	0.98858
7	1–12	0.98955

**Figure 19:**
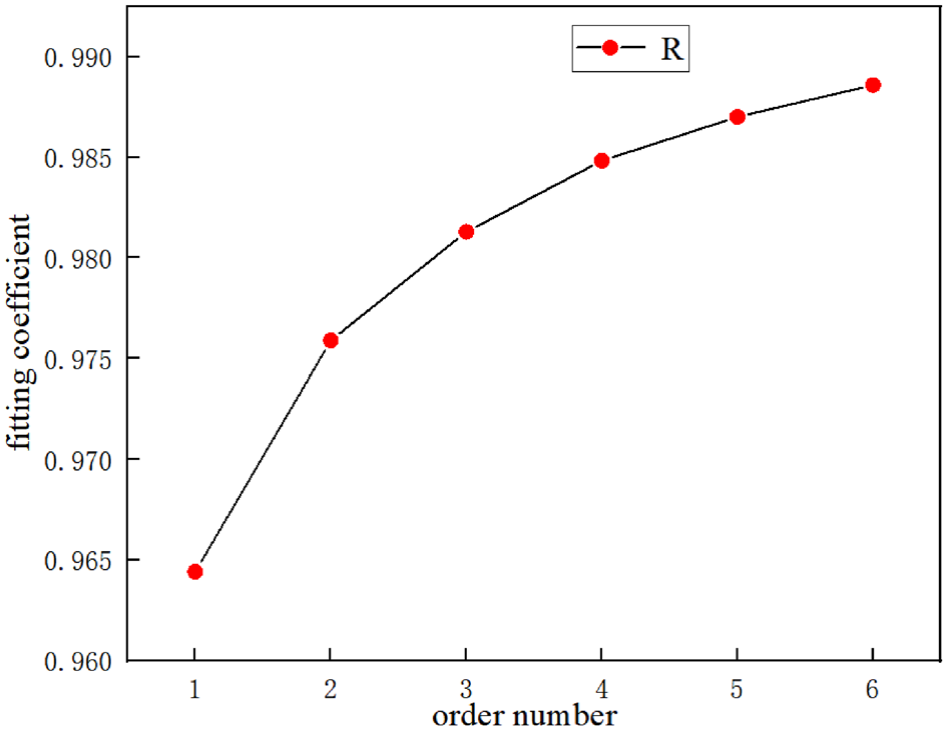
The relationship between the number of experiments and the fitting coefficient.

**Figure 20:**
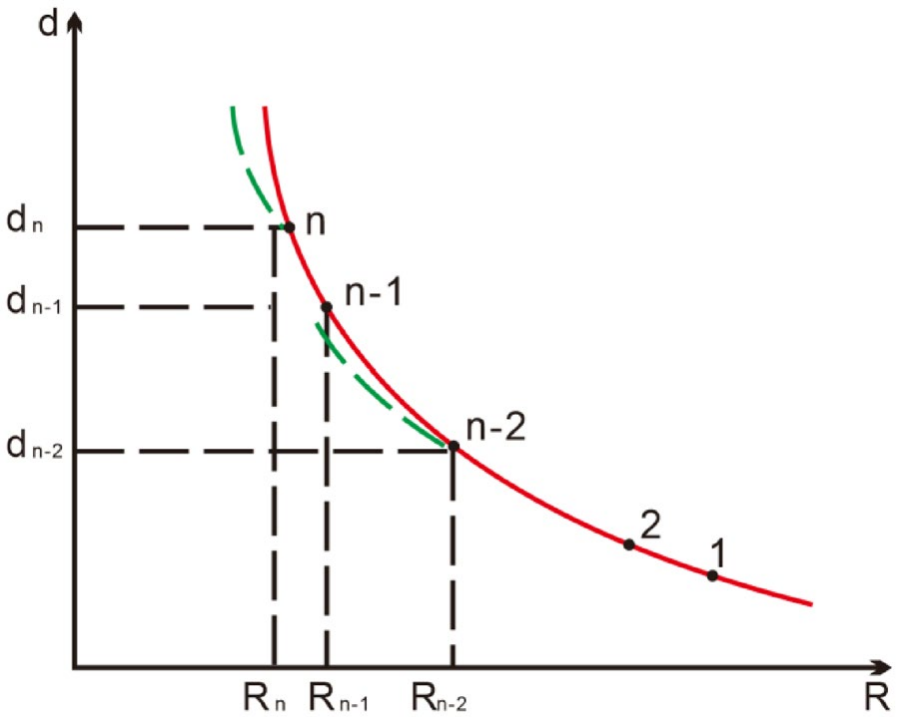
Control flow.

As shown in Fig. 19, as the number of experiments increased, the degree of fitting increased. Based on this, an adaptive control model is proposed, and because there is a one-to-one correspondence between the forming radius R and the lateral displacement d of the profile during the profile rolling process, we believe that a rolling forming curve exists during the profile rolling process. This curve reflects the forming trend of the profile, and if it is fitted, it can be used for the profile rolling forming process, as shown in Fig. 20.

Suppose that the workpiece has been rolled $\left( {{\text{n}} - 2} \right)$ times, and $\left( {{\text{n}} - 2} \right)$ sets of data have been obtained. Regression calculation of the $\left( {{\text{n}} - 2} \right)$ sets of R and d data can yield an $\left( {{\text{R}} - {\text{d}}} \right)_{{{\text{n}} - 2}}$ curve, which is represented by the green line in the figure. $\left( {{\text{R}} - {\text{d}}} \right)_{{{\text{n}} - 2}}$ the roll bending forming curve can be used as the control curve to guide the next roll bending (i.e. $\left( {{\text{n}} - 1} \right)$ times), and a group of R and d data can be obtained after $\left( {{\text{n}} - 1} \right)$ times of rolling and bending. A more practical $\left( {{\text{n}} - 1} \right)$ forming curve can be obtained by regression calculation of the increased $\left( {{\text{R}} - {\text{d}}} \right)_{{{\text{n}} - 1}}$ groups of R and d data, which can achieve more accurate profile bending control.

## Conclusion

Based on the problems existing in the original hydraulic and electro-hydraulic systems, a more advanced servo electric cylinder was adopted as the solution. The servo electric cylinder does not require a dedicated hydraulic station to provide power, so an integrated bed can be designed to make the workbench more stable. In terms of profile forming control, an adaptive control model was established based on experimental considerations. After verification, this model can be used as a mathematical model in the preliminary forming control of profile rolling bending.

1. Verified the rationality of the application of servo electric cylinder in the bending machine. In terms of mechanical structure and type selection, the left and right lower rollers of the machine are driven by servo-electric cylinders, with a position control accuracy of ± 0.1 mm and high repeatability accuracy to ensure the consistency of the profile processing, and the fast response of the servo-electric cylinder position control ensures the accuracy of the profile bending. Spherical bearings are used at the push rod connection, which can withstand radial loads, axial loads or combined radial and axial loads at the same time. The rollers were mounted on a specially designed T-joint table to ensure a smooth feeding and machining.
2. By combining the VS software with the GTS-800 motion controller, the PC can be turned into a motion controller with real-time motion control capability. The control scheme selected in this study is more modular, hierarchical, and standardized compared to traditional control schemes.
3. The actual R and d values of the four-roller CNC bending machine are utilized, and curve fitting method is employed to select a basic curve equation as the foundation for the control model in the automatic control design. Coefficient of determination $R$ = 0.98955,high degree of linear regression fitting. The average error of. The five groups of tests were 1.834%, which can be used for the preliminary processing of the profiles. As the number of tests increases, accurate processing of profiles can be achieved. Compared with pure theoretical derivation models, this scheme has stronger universality. To sum up, it can be seen as a major improvement and upgrading of the four-roll bending machine.

## Data Availability

The datasets used and/or analysed during the current study available from the corresponding author on reasonable request.

## References

[1] Yu J, Zhao G, Chen L (2016). Investigation of interface evolution, microstructure and mechanical properties of solid-state bonding seams in hot extrusion process of aluminum alloy profiles. J. Mater. Process. Technol..

[2] Lou S, Zhao G, Wang R, Xianghong W (2008). Modeling of aluminum alloy profile extrusion process using finite volume method. J. Mater. Process. Technol..

[3] Mercuri, A. *et al.* Experimental and numerical analysis of roll bending process of thick metal sheets. In *IOP Conference Series: Materials Science and Engineering* 012067 (IOP Publishing, 2021).

[4] Fu Z, Mo J, Zhang W (2009). Study on multiple-step incremental air-bending forming of sheet metal with springback model and FEM simulation. Int. J. Adv. Manuf. Technol..

[5] Paralikas J, Salonitis K, Chryssolouris G (2011). Investigation of the effect of roll forming pass design on main redundant deformations on profiles from AHSS. Int. J. Adv. Manuf. Technol..

[6] Feng, Z. & Champliaud, H. Comparison between numerical simulation and experimentation of asymmetrical three-roll bending process. In *ASME International Mechanical Engineering Congress and Exposition* 33–37 (2010)

[7] Wenzheng L, Wenfeng H, Weijun W (2017). Common faults and countermeasures of hydraulic cylinders in the bottom mounted HAGC system of four high rolling mills. Modern Commer. Ind..

[8] Yinhuang Li (2005). Introduction to intelligent electric actuators and their development trends. Gansu Chem. Ind..

[9] Mingying Y, Qiang L, Yanyan L (2013). Force analysis and drive power calculation of four roll plate rolling machine. Forg. Technol..

[10] Yuling H, Weiqiang W, Jianfeng C (2018). Optimization design of transmission connection plate for four roll plate rolling machine. Mech. Res. Appl..

[11] Jiuhua Z, Xin C, Wang Xinyu Wu, Kai HJ (2018). Deformation analysis and compensation technology of the lower roll of a four roll plate rolling machine. Forg. Technol..

[12] Jiujun Y, Tongke L (2012). Design of a fully hydraulic four roll bending machine. Yizhong Technol..

[13] Yang X, Liu Y, Yue M (2019). Mechanical research on working rolls in four-roll plate prebending process. J. Plast. Eng..

[14] Wang Yan Hu, Guanghui JX, Jianbo W, Shusheng Li (2016). Mechanism and experimental analysis of four roll pre bending and continuous roll bending. J. Plast. Eng..

[15] Chen XD, Cai ZY, Li MZ (2003). Numerical simulation of springback of multi-point forming for sheet meta-l without blankholders. J. Plastic Engineer.

[16] Zhan M, Yang H, Huang L (2006). Springback analysis of numerical control bending of thin-walled tube using numerical-analytic method. J. Mater. Process. Technol..

[17] Panthi SK, Ramakrishnan N, Pathak KK (2007). An analysis of springback in sheet metal bending using finite element method (FEM). J. Mater. Process. Technol..

[18] Yu Q, Tong Y (2011). Overview of profile roll-bending forming and defect compensation methods. Hydraul. Pneumat. Seal..

[19] Hua M, Baines K, Cole IM (1995). Bending mechanisms, experimental techniques and preliminary tests for the continuous four-roll plate bending process. J. Mater. Process. Technol..

[20] Shu D, Wang C (1994). Discussion on the mathematical model of automatic control of three-roll bending machine. Shipbuild. Technol..

[21] Sen Li, Fulin C, Bin Li (2011). Calculation of side roller displacement during bending of a four roller plate bending machine. Forg. Technol..

